# Features of the Human Antibody Response against the Respiratory Syncytial Virus Surface Glycoprotein G

**DOI:** 10.3390/vaccines8020337

**Published:** 2020-06-25

**Authors:** Kristina Borochova, Katarzyna Niespodziana, Katarina Stenberg Hammar, Marianne van Hage, Gunilla Hedlin, Cilla Söderhäll, Margarete Focke-Tejkl, Rudolf Valenta

**Affiliations:** 1 Division of Immunopathology, Department of Pathophysiology and Allergy Research, Center for Pathophysiology, Infectiology and Immunology, Medical University of Vienna, 1090 Vienna, Austria; kristina.borochova@meduniwien.ac.at (K.B.); katarzyna.niespodziana@meduniwien.ac.at (K.N.); margarete.focke-tejkl@meduniwien.ac.at (M.F.-T.); 2 Department of Women’s and Children’s Health, Karolinska Institutet, 171 77 Stockholm, Sweden; Katarina.Stenberg@bsmartina.se (K.S.H.); gunilla.hedlin@ki.se (G.H.); cilla.soderhall@ki.se (C.S.); 3 Astrid Lindgren Children’s Hospital, Karolinska University Hospital, 14186 Stockholm, Sweden; 4 Centre of Allergy Research, Karolinska Institutet, 171 77 Stockholm, Sweden; 5 Division of Immunology and Allergy Unit, Department of Medicine, Solna, Karolinska Institutet and Karolinska University Hospital, 171 77 Stockholm, Sweden; marianne.van.Hage@ki.se; 6 NRC Institute of Immunology FMBA of Russia, 115478 Moscow, Russia; 7 Laboratory for Immunopathology, Department of Clinical Immunology and Allergy, Sechenov First Moscow State Medical University, Moscow 119991, Russia; 8 Karl Landsteiner University of Health Sciences, 3500 Krems, Austria

**Keywords:** respiratory syncytial virus (RSV), glycoprotein protein, recombinant proteins, G protein, epitope, antibody response

## Abstract

Respiratory syncytial virus (RSV) infections are a major cause of serious respiratory disease in infants. RSV occurs as two major subgroups A and B, which mainly differ regarding the surface glycoprotein G. The G protein is important for virus attachment and G-specific antibodies can protect against infection. We expressed the surface-exposed part of A2 strain-derived G (A2-G) in baculovirus-infected insect cells and synthesized overlapping peptides spanning complete A2-G. The investigation of the natural IgG response of adult subjects during a period of one year showed that IgG antibodies (i) recognize G significantly stronger than the fusion protein F0, (ii) target mainly non-conformational, sequential peptide epitopes from the exposed conserved region but also buried peptides, and (iii) exhibit a scattered but constant recognition profile during the observation period. The IgG subclass reactivity profile (IgG_1_ > IgG_2_ > IgG_4_ = IgG_3_) was indicative of a mixed Th1/Th2 response. Two strongly RSV-neutralizing sera including the 1st WHO standard contained high IgG anti-G levels. G-specific IgG increased strongly in children after wheezing attacks suggesting RSV as trigger factor. Our study shows that RSV G and G-derived peptides are useful for serological diagnosis of RSV-triggered exacerbations of respiratory diseases and underlines the importance of G for development of RSV-neutralizing vaccines.

## 1. Introduction

Respiratory syncytial virus (RSV) infections are a major cause of severe respiratory tract diseases in infancy that may have a fatal outcome [1]. RSV infections induce only an incomplete immunological protection and therefore occur as recurrent diseases throughout life [2]. Accordingly, RSV affects people of all ages and RSV infections are suspected to be trigger factors for severe respiratory illnesses also in adults and elderly people [3]. Some studies even provide evidence that certain children who had experienced severe RSV infections early in life may become predisposed to develop asthma later [4].

RSV was first described as a cause of respiratory illness in 1955 in chimpanzees and later was found to be responsible for bronchiolitis in infants [5,6]. The envelope of RSV contains the small hydrophobic (SH) protein, the fusion protein (F), and the attachment glycoprotein (G) [7]. The two antigenic subgroups of RSV, RSV-A, and RSV-B, can be distinguished based on sequence variations in the attachment (G) glycoprotein, which shows sequence variability, whereas the sequence of fusion protein F is highly conserved [8,9,10]. The determination of the nucleotide and deduced amino acid sequence of the RSV G protein showed that it is a viral membrane protein, which is different from many other viral membrane proteins regarding its architecture [11]. In fact, G is a highly glycosylated protein consisting of a N-terminal cytoplasmic domain, followed by a hydrophobic transmembrane domain and two mucin-like regions, which surround a central conserved disulphide-bonded noose that protrudes from the virus [7]. Glycosylation is not essential but seems to contribute to surface expression of the G protein [12]. Interestingly, RSV G is produced as a membrane-associated form and as a secreted form, which may serve as an antigenic decoy for the host’s immune system. Much attention has been focused on the RSV fusion protein F because it is the target for a monoclonal therapeutic antibody, palivizumab, which currently is the only available specific treatment for RSV infections [13,14]. However, the RSV glycoprotein G is also very important because it has been identified as the attachment protein of the virus, which makes the initial contact to the host cells. Furthermore, several studies have identified the G protein as a relevant target for the antibody response of infected subjects. Since several G-specific antibodies were found to exhibit virus-neutralizing activity, the protein is currently reconsidered as a possible candidate molecule for the development of RSV-specific vaccines [15,16,17,18,19].

Results regarding the natural human RSV-specific antibody response in infected and/or RSV-exposed subjects are not yet complete and partially controversial. A study using purified RSV G and F proteins found that more infants with RSV bronchiolitis had antibodies against the G than to F protein [20], two other studies reported similar antibody responses against F and G [21,22], whereas yet two other studies identified F as a major target for human antibody responses [23,24]. The presence of conformational as well as of sequential epitopes on F and G has been reported [25]. Likewise, information regarding the IgG subclass recognition of G is controversial. Some studies suggest a predominant IgG_1_ and IgG_2_ anti-G response [22,23], others report a dominance of IgG_1_ and IgG_3_ responses [21], which both would be indicative of a Th1-type immune response [26,27]. However, even formation of RSV-specific IgE, which would be suggestive of a Th2-like RSV-specific immune response has been reported [24]. The occurrence of IgG epitopes in the central conserved region of RSV G [28,29] and at the C-terminus [30] has been demonstrated but no detailed epitope mapping of the human IgG response against RSV G has been performed so far.

In order to investigate the specific antibody response against RSV G in detail, we expressed the soluble forms of A2 strain-derived G in insect cells and produced a set of overlapping peptides spanning the complete A2 strain-derived G, including the intracellular and transmembrane domain. Furthermore, we compared the magnitude of the antibody response against A2 strain-derived G with that of a recombinant palivizumab-reactive A2-derived F0. Our study shows that the natural RSV G-specific IgG levels in healthy adult humans are significantly higher than the ones against F0 and suggest the G protein and its sequential epitopes as useful tools for the serological measurement of RSV-specific antibody responses in the assessment of RSV-triggered respiratory illness.

## 2. Materials and Methods

### 2.1. Construct Design, Expression, and Purification of Soluble Recombinant G Proteins

**Figure 1:**
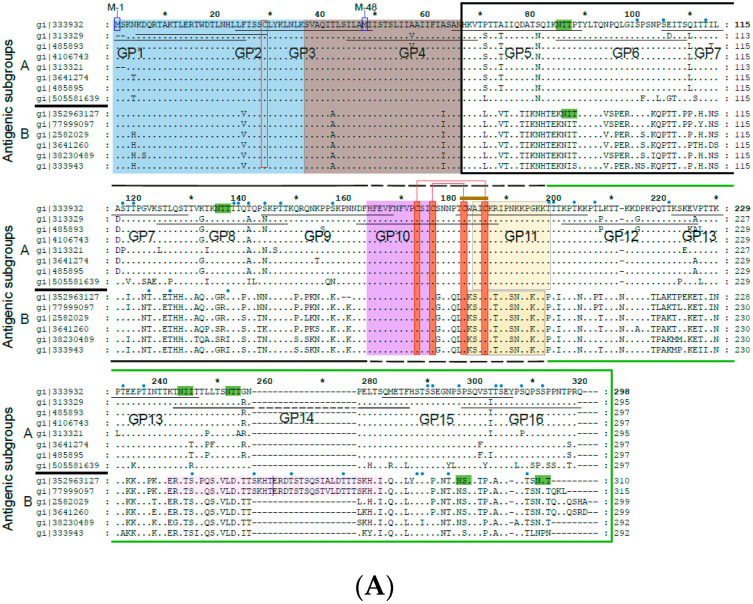

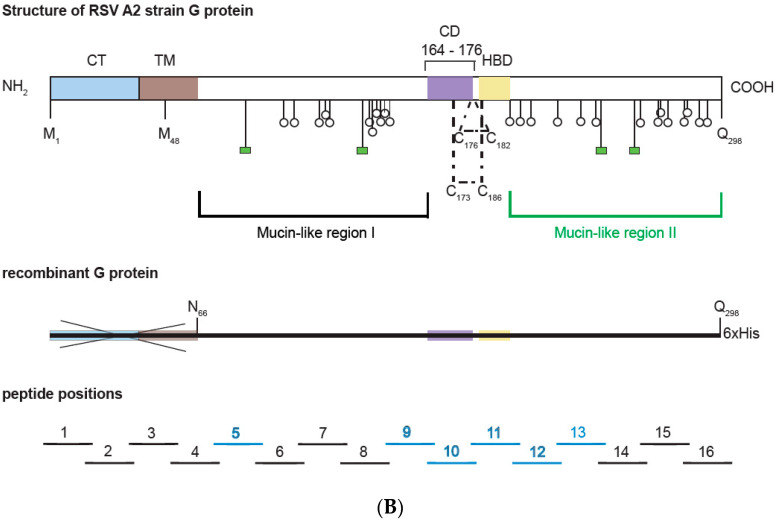
(**A**) Alignment of the amino acid sequences of G proteins from distantly related strains from subgroup A and B. Subgroup A and B sequences as indicated on the left margins (gene identifier-gi-numbers indicated) were aligned with the G protein sequence from A2 strain (subgroup A) on the top. Dots show identical amino acids and dashes indicate gaps. The membrane-anchored form (Gm) starts with methionine M-1 and the secreted form (Gs) with M-48. The two mucin-like regions are indicated by a black (mucin-like region I) and by a green (mucin-like region II) frame. Conserved cysteine residues are denoted by red boxes and predicted N- and O-linked glycosylation sites by highlighting the consensus sequence NXT/S in green and with small blue ovals, respectively. A CX3C-like motif similar to one in the chemokine CX3CL1 (fraktalkine) is indicated by a brown bar in the Cysteine noose which is stabilized by two disulfide bonds in the center of the protein. The N-terminal cytoplasmic tail (CT) is boxed in blue, the hydrophobic transmembrane anchor (TM) in brown, the Heparin-binding domain (HBD) in pale orange and the central conserved domain in green. The pink regions in subgroup B RSV strains are duplicated. Synthetic peptides (GP1–GP16) used for epitope mapping spanning the G protein sequence are indicated by black horizontal lines. (**B**) Overview of the G protein structure showing the different domains and N- as well as O-linked glycans. Below, the recombinant G protein construct and overlapping peptides 1–16 (IgG-reactive peptides in blue) are indicated. Blue * represents the Cytoplasmic Tail and is described in figure legends.

**Figure 2:**
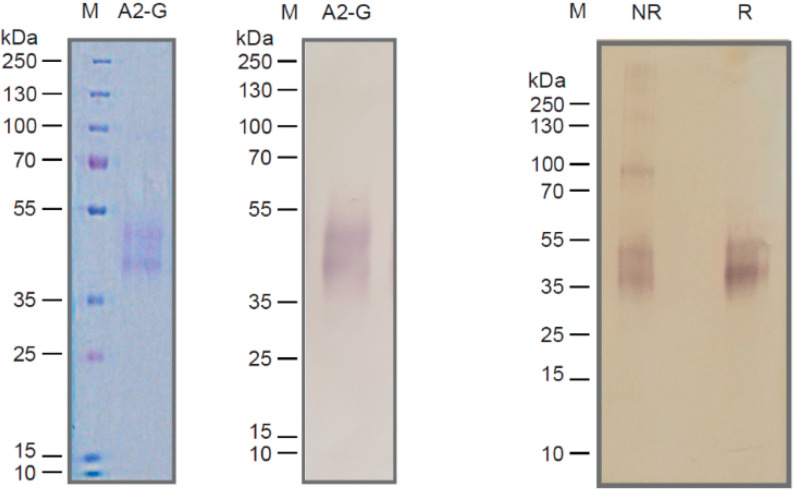
Characterization of recombinant insect cell-expressed G protein. Coomassie-blue stained SDS-PAGE with recombinant A2-G and a molecular-weight marker (kDa: kilo Dalton) (left). Corresponding immunoblot stained with an anti-His-tag antibody (middle). Immunblotted A2-G separated under non-reducing (lane NR) or reducing conditions (lane R) stained with anti-His antibodies. Molecular weights (kDa) are indicated on the left margins.

**Figure 3:**
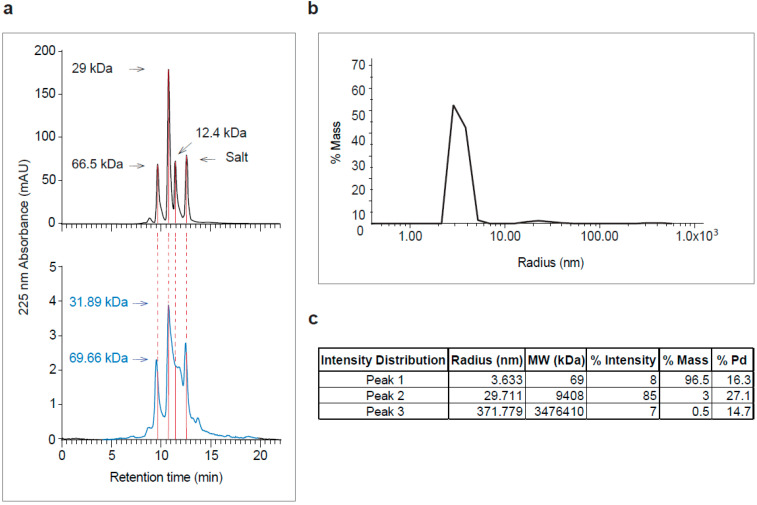
Analysis of purified recombinant A2-G by size exclusion chromatography (SEC) and dynamic light scattering (DLS). (**a**) Elution profiles of molecular weight standards (top) and of purified A2-G (bottom, in blue). Absorbances at 225 nm (y-axes) are shown for different retention times (y-axes) with indicated molecular weights in kDa. (**b**) DLS profile of purified A2-G (y-axis: % mass; *x*-axis: radius-nm) are shown. (**c**) Summary of peak features (radius, molecular weight, % intensity, % mass and % Pd).

The amino acid sequence of the A2-derived attachment glycoprotein was retrieved from UniProtKB/Swiss-Prot-Expasy (accession number: P03423.1). The cDNA coding for the extracellular domain of the G protein (amino acids 66 to 298) (Figure 1) was codon optimized for insect cell expression and synthesized including a sequence coding for a C-terminal hexahistidine tag (ATG: biosynthetics, Merzhausen, Germany). The DNA construct was cloned into the BamHI/SmaI sites of the pTM1 vector, harboring the baculoviral polyhedron promoter sequence [31]. The identity of the construct was confirmed by BamHI/SmaI single and double restriction analysis of midi-prep plasmid DNA (Promega, Madison, WI, USA) followed by DNA sequencing (VBC-Biotech Service, Vienna, Austria). Baculovirus amplification was performed in the Spodoptera frugiperda (Sf9) insect cell line, whereas protein expression was done in 20 mL Trichoplusia ni (High Five) insect cell cultures at 27 °C and 80 rpm for two days as described by Pahr et al. [32]. Sf9 and High Five cells were obtained from Life Technologies (Carlsbad, CA, USA). Recombinant His-tagged A2-G was purified by affinity chromatography using a Hi60 Ni-NTA resin (Clontech Laboratories, Inc.; Takara Biotechnology; Fitchburg, WI, USA) under native buffer conditions with 100 to 200 mM imidazole concentrations for protein elution (Qiagen, Hilden, Germany) (Figure 2 and Figure 3). Pure glycoprotein fractions were pooled and dialysed in Spectra/Por dialysis membrane tubes, cut-off of 12-14 kDa (Spectrum Laboratories, Inc., Rancho Dominguez, CA, USA), against the final storage buffer (0.137 M NaCl, 2.7 mM KCl, 8 mM Na_2_HPO_4_, 2 mM KH_2_PO_4_, pH 7.2). The purity of the protein was evaluated by 12% SDS-PAGE followed by Coomassie-staining and protein identity was confirmed by immunoblotting using a 1:1000 diluted monoclonal mouse anti-His-tag antibody (Dianova, Hamburg, Germany). Bound antibodies were detected with 1:1000 diluted alkaline phosphatase-coupled rat anti-mouse IgG antibodies (BD Bioscience/Pharmingen, Heidelberg, Germany). The protein concentration was determined in micro-plate format using a BCA Protein Assay Kit (Pierce, Rockford, IL, USA). The pre-stained protein molecular weight marker (PageRuler prestained Protein Ladder Plus, Fermentas, St Leon-Rot, Germany) was used as a standard in SDS-PAGE.

### 2.2. Size Exclusion Chromatography and Dynamic Light Scattering

For size exclusion chromatography, 0.1 mL of purified A2-G protein (0.12 mg/mL in 0.137 M NaCl, 2.7 mM KCl, 8 mM Na_2_HPO_4_, 2 mM KH_2_PO_4_, pH 7.2) was loaded onto a BioSeb SEC-S 3000 column (300 × 7.80 mm; Phenomenex, Aschaffenburg, Germany) at 4 °C, equilibrated with protein storage buffer (0.137 M NaCl, 2.7 mM KCl, 8 mM Na_2_HPO_4_, 2 mM KH_2_PO_4_, pH 7.2). The flow rate was 0.5 mL min^−1^ and chromatograms were obtained at 225 nm (UV-VIS) wavelength. The molecular mass of defined peaks of A2-G were calculated based on the elution profile of a gel filtration protein standard (66 kDa: Bovine serum albumin (1 mg); 29 kDa: Carbonic Anhydrase (1 mg); 12.4 kDa: Cytochrome C (1 mg); Sigma Aldrich, Taufkirchen, Germany) performed under identical buffer conditions.

Dynamic light scattering (DLS) was performed on a DynaPro-NanoStar device using 20 µL of 0.1 mg/mL A2-G protein in a disposable micro cuvette (Wyatt Technology; Hollister Avenue, Santa Barbara, CA, USA) at 20 °C in 0.137 M NaCl, 2.7 mM KCl, 8 mM Na_2_HPO_4_, 2 mM KH_2_PO_4_, pH 7. Twenty acquisitions, with a five-second acquisition time for each measurement, were carried out with a laser power of 50%. Hydrodynamic radius was calculated using dynamics V6 software (Wyatt Technology Corporation, Santa Barbara, CA, USA).

### 2.3. Denaturation and Deglycosylation of Recombinant A2-G for Enzyme-Linked Immunosorbent Assay (ELISA) Experiments

Coating of ELISA plates with denatured and reduced or non-reduced A2-G protein was carried out with native A2-G in coating buffer (0.05 M carbonate-bicarbonate, pH 9.6) plus 0.46% SDS with or without 15 mM TCEP (Tris (2-carboxyethyl) phosphine hydrochloride). Protein samples were pre-heated up to 95 °C for 10 min before application to the ELISA plate.

Enzymatic deglycosylation was performed with PNGase F (New England Biolabs, Ipswich, USA) by overnight incubation at 37 °C according to the manufacturer’s instructions and then the A2-G was coated on the ELISA plates.

### 2.4. Peptide Synthesis and Purification

Sixteen overlapping peptides with a length of 25 to 27 amino acids spanning the complete A2 RSV strain G amino acid sequence (UniProtKB/Swiss-Prot-Expasy; accession number: P03423.1) were produced by solid-phase synthesis with the 9-fluorenyl-methoxy-carbonyl-method, using TentaGel preloaded resins (e.g.,: TentaGel™ S PHB-Arg(Pbf) Fmoc; Sigma-Aldrich, Vienna, Austria) and a microwave Peptide Synthesizer CEM-Liberty instrument (CEM Corp., Matthews, NC, USA) as described by Focke-Tejkl M., et al. (Table 1) [33]. Following peptide purification by reversed-phase HPLC, using Dionex HPLCUltiMate 3000 system (Thermo Fisher Scientific, Inc., Vienna, Austria), the identity and mass of each synthetic peptide was confirmed by MALDI-TOF analysis (Microflex, MALDI-TOF, Bruker Daltonics, Billerica, MA, USA) using a CA matrix (α-Cyano4-hydroxycinnamic acid dissolved in 70% acetonitrile (ACN), 0.1% trifluoroacetic acid (TFA)). For the sample preparation, a 1:1 mixture of peptide sample and matrix solution was used, which was deposited onto a target and air dried. Acquired spectra were analysed with the Bruker Daltonics FlexAnalysis software. Pure peptide fractions with the correct molecular weight were pooled and lyophilized using CHRiST-Alpha 2-4 LSC Lyophilizator (SciQuip; Newtown, Wem, Shropshire, UK). Purified and lyophilized peptides samples were dissolved in sterile ddH_2_O before use for ELISA experiments.

### 2.5. Demographic Characterization of Study Subjects

**Figure 4:**
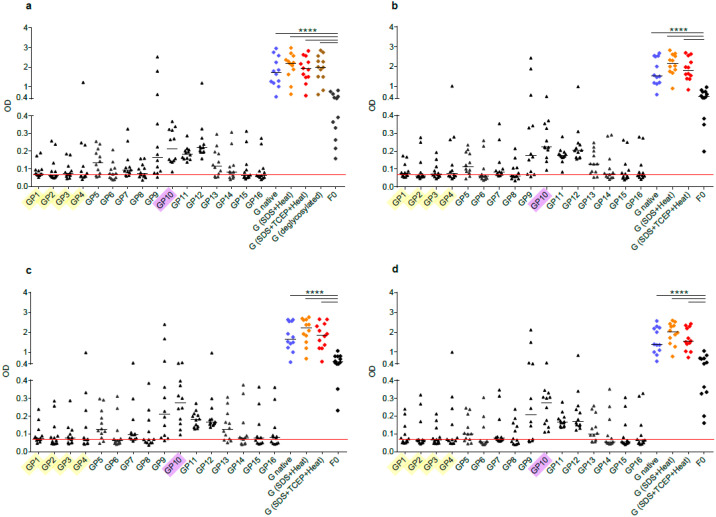
Human antibody responses to recombinant native G, denatured and deglycosylated G and G-derived synthetic peptides. Shown are IgG levels (y-axes: optical density values) in sera from 12 healthy adult individuals obtained at different time points ((**a**) January-February; (**b**) April-May; (**c**) August-September; (**d**) January-February next year) to recombinant, native G (blue), G denatured by heat and SDS (orange), G denatured by heat, SDS and TCEP (red), deglycosylated G (only for time point a, brown), G-derived peptides (GP1-GP16; buried peptides: yellow; peptides without glycosylation sites: pink) and recombinant F0 (x-axes). Horizontal lines within scatter plots indicate median values. The cut-off (mean of buffer control plus three times standard deviation) is indicated by horizontal red lines. Significant differences between G and F0-specific antibody levels are indicated (**** *p* < 0.0001).

**Figure 5:**
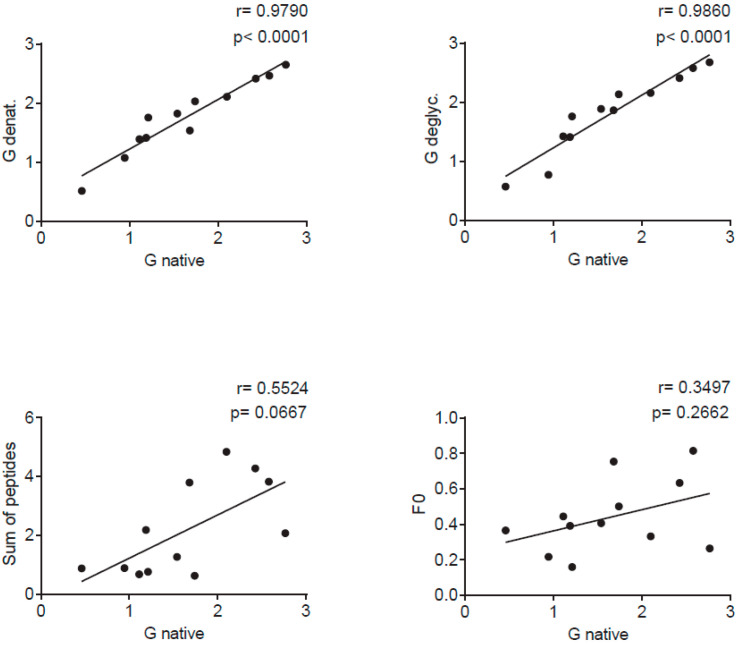
Correlations of IgG levels specific for native G (x-axes) and denatured G (SDS, TCEP and heat) (*y*-axis) (**upper left**), deglycosylated G (*y*-axis) (**upper right**), the sum of peptide-specific IgG (*y*-axis) (**lower left**) and for F0 (*y*-axis) (**lower right**) measured in sera from 12 adult individuals in individual scatter plots with Spearman correlation coefficient r and p values.

Serum samples from adult subjects were from the serum bank of the Division of Immunopathology, Department of Pathophysiology and Allergy Research at the Medical University of Vienna, Austria. They were used for the individual experiments according to available volumes and included a set of sera from 12 healthy adult subjects (n = 12; males: 5; females: 7; age range in years: 26–46; mean age in years: 32; allergic subjects: 2; non-allergic subjects: 10.) and had been collected during a period of one year at four different time points (January/February, April/May, September, January/February) which were used for the experiments described in Figure 4 and Figure 5, Figures S1 and S2.

**Figure 6:**
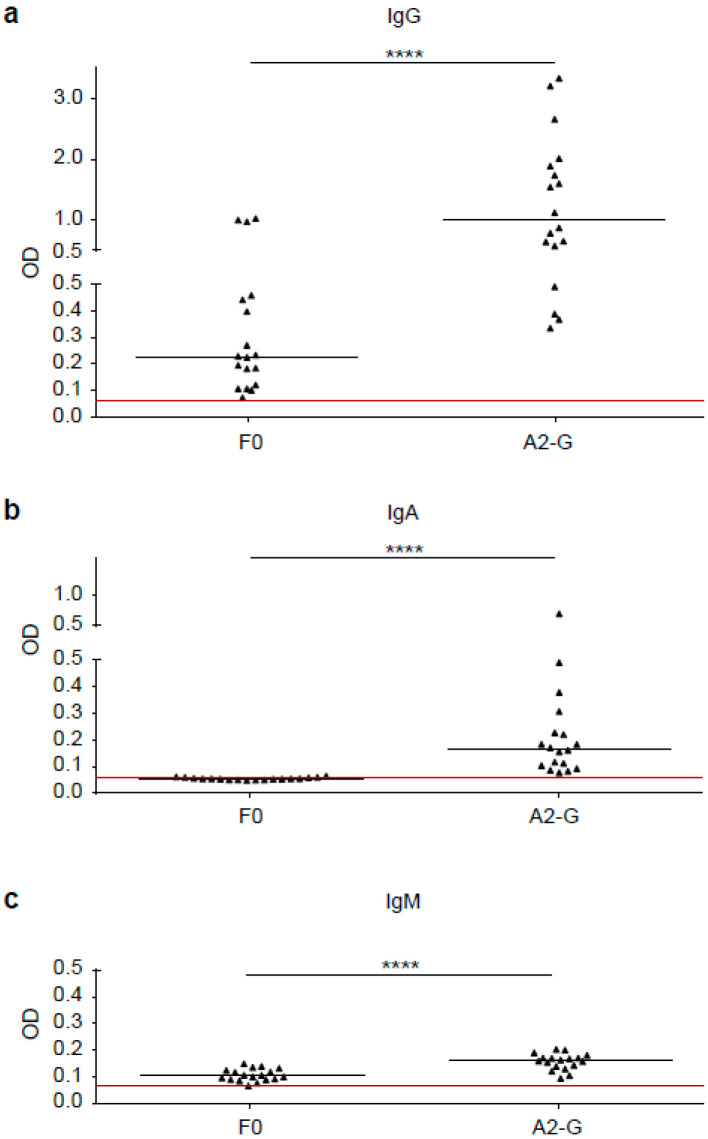
Comparison of G and F0-specific human IgG, IgM and IgA responses. Shown are IgG (**a**), IgA (**b**) and IgM (**c**) levels (y-axes: optical density OD values) against A2-G and F0 measured by ELISA in sera from 18 healthy adult individuals. Horizontal lines within scatter plots indicate median values. The cut-off (mean of buffer control plus three times standard deviation) is indicated by horizontal red lines. Significant differences between G and F0-specific antibody levels are indicated (**** *p* < 0.0001).

**Figure 7:**
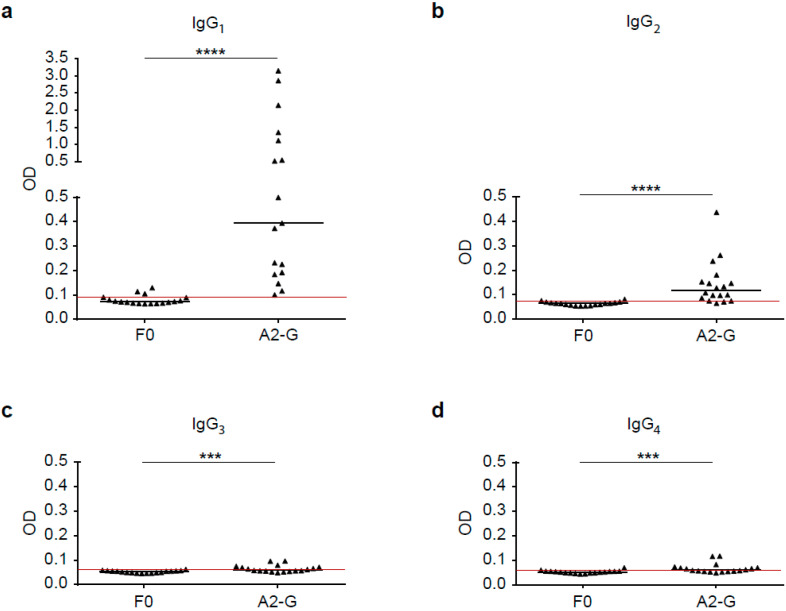
Human IgG subclass responses to recombinant native G and F0. Shown are IgG subclass levels, IgG_1_ (**a**), IgG_2_ (**b**), IgG_3_ (**c**) and IgG_4_ (**d**) in sera from 18 healthy adult individuals to recombinant, native G and F0 (x-axes), (y-axis: optical density values; IgG_1_, IgG_2_, IgG_3_ and IgG_4_). Horizontal lines within scatter plots indicate median values. The cut-off (mean of buffer control plus three times standard deviation) is indicated by horizontal red lines. Significant differences between G and F0-specific antibody levels are indicated (*** *p* < 0.001; **** *p* < 0.0001).

For the experiments described in Figure 6 and Figure 7, sera from 18 healthy adult subjects were randomly picked (n = 18; males: 9; females: 9; age range in years: 23–59; mean age in years: 40.4; allergic subjects: 7; non-allergic subjects: 11). In the IgE dot-blot experiment, in Figure S3, a set of sera from 15 adult subjects were used (n = 15; males: 7; females: 8; age range in years: 20–51; mean age in years: 34.6; allergic subjects: 8; non-allergic subjects: 7).

Serum samples from Swedish preschool children who had experienced respiratory virus-induced wheezing attacks were used [34,35]. We included sera from 12 children (obtained at the day of admission (acute visit and at follow-up visits approximately 11 weeks later; n = 12; males: 8; females: 4; age range in months: 6–34; mean age in months:19) with a positive PCR test results for RSV in nasal swab samples. Table 2 provides an overview of the sera used for the different experiments.

One serum from an adult person with potent virus neutralization activity was also used to assess G-specific antibody reactivity. Furthermore, we included the standard antiserum for RSV (1st International Standard for Antiserum to RSV, WHO International Standard, National Institute for Biological Standards and Control (NIBSC) code: 16/284 which was obtained from NIBSC, Potters Bar, Hertfordshire, EN6, 3QG, UK) and the serum from a subject with high virus neutralization capacity.

Pseudo-anonymized sera were analyzed with approval from the ethics committee of the Medical University of Vienna (EK1641-2014). Informed written consent was obtained from all adult subjects from the Austrian Center. Regarding the Swedish children, the samples were also pseudo-anonymized and written informed consent was obtained from the parents or by the legal guardians and the study was approved by the Regional Ethics Committee of Karolinska Institutet, Stockholm, Sweden.

### 2.6. Enzyme-Linked Immunosorbent Assay (ELISA) Experiments

**Figure 8:**
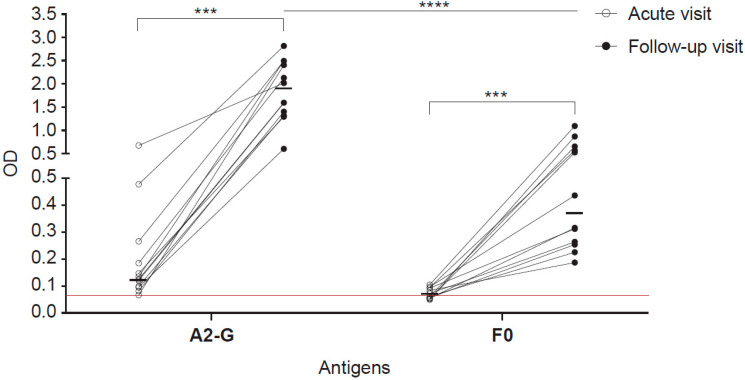
IgG responses specific for G and F0 in children with an acute wheezing attack at base-line and in follow-up blood samples. Shown are optical densities (ODs) corresponding to IgG levels specific for A2-G and F0 determined in sera from children attending emergency care at base line (open circles) and in follow-up samples collected several weeks after (black circles). The cut-off (mean of buffer control plus three times standard deviation) is indicated by horizontal red lines. Horizontal lines within the scatter plots indicate median values. Depicted are 12 RSV-positive children according to PCR test. Comparative tests were non-parametric paired (Wilcoxon matched-pairs signed rank test) or unpaired test (Mann-Whitney U test) as appropriate. Significant differences of antibody levels between base-line (acute visit) and follow-up samples are indicated (*** *p* < 0.001, **** *p* < 0.0001).

**Figure 9:**
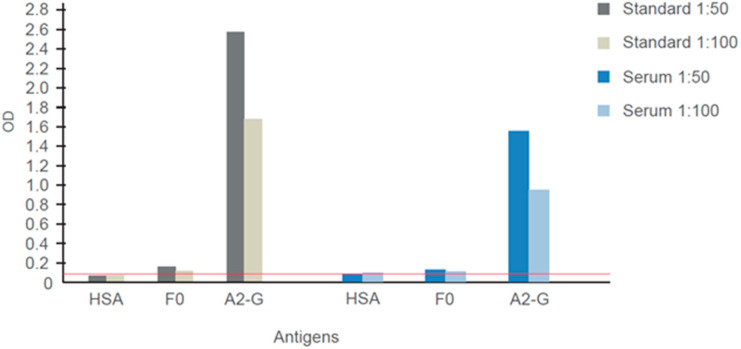
IgG responses specific for G and F0 determined in the WHO 1st International Standard for Antiserum to Respiratory Syncytial Virus and another virus-neutralizing human serum. Shown are means of optical density (OD) values (< 5% deviation) corresponding to IgG levels specific for G, F0 and human serum albumin determined for different dilutions (1:50, 1:100). The cut-off (mean of buffer control plus three times standard deviation) is indicated by a horizontal red line.

Specific IgG levels in sera were determined by direct ELISA. Native A2-G protein, heat denatured and reduced A2-G (coating buffer, 0.46% SDS, 15 mM TCEP), heat denatured A2-G (coating buffer, 0.46% SDS), enzymatically deglycosylated A2-G, A2-G derived peptides and F0 were coated at a concentration of 2 µg/mL, in coating buffer (0.05 M sodium-bicarbonate, pH 9.6) overnight at 4 °C to ELISA plates (Microplate, 96 well, PS, half area, clear and medium binding; Greiner bio-one, Frickenhausen, Germany). In all ELISA experiments, HSA (human serum albumin) was coated for control purposes at the concentration of 2 µg/mL. After washing with PBS/T (1× PBS and 0.05% *v/v* Tween 20) and blocking with BSA (2% *w/v* BSA, in 1× PBS/T) for 5 h at room temperature, patient serum dilutions (1:50 for experiments described in Figure 4 and Figure 5, Figures S1 and S2, Figure 8 and for Figure 6 upper panel; for Figure 9 1:50 and 1:100 dilutions were used) were added and incubated overnight at 4 °C. After washing the plates, bound IgG was detected with horseradish peroxidase (HRP)-conjugated anti-human-IgG (1:5000, BD Pharmingen, HRP anti-human IgG). For IgA, IgM and IgG subclasses experiments plates were coated with antigens and blocked as described above. Patient serum dilution (1:40) was incubated overnight at 4 °C. Bound antibodies were detected with mouse anti-human IgG_1_, IgG_2_, IgG_4_, IgA, IgM (1:1000, BD Pharmingen, San Diego, CA, USA) or mouse anti-human IgG_3_ (1:2500, Sigma Aldrich, St. Louis, MO, USA) for 2 h at room temperature. After another plate washing step bound antibodies were determined by horseradish-peroxidase (HRP)-coupled sheep anti-mouse IgG (1:2000, Amersham Bioscience, Freiburg, Germany) incubated for one hour at room temperature. [36] After a final plate washing step, the colour reaction was started by adding 50 µl/well of substrate solution (200 mg 2,2’-azino-bis(3-ethylbenzothiazoline-6-sulphonic acid (ABTS); Sigma-Aldrich; in 200 mL citric buffer (61.5 mM citric acid, 77.3 mM Na2HPO4 × 2 H_2_O, pH 4) and 20 µL hydrogen peroxide). The optical density (OD) values corresponding to the levels of antigen-specific antibodies were determined at 405 and 492 nm in an ELISA reader (Perkin Elmer EnSpire 2300 multilabel reader, 940 Winter Street, Waltham, MA, USA or TECAN infinite F50, Maennedorf, Switzerland). Buffer controls without addition of serum were included on each plate to determine cut-off levels for background. All determinations were conducted as duplicates, with a variation of less than 5% and results were expressed as normalized mean values. A plate to plate normalization was conducted by including a reference serum on each of the plates as described by Stern et al. [27].

### 2.7. Virus Neutralization Assay

The RSV neutralization assays were performed by Viroclinics (Viroclinics Biosciences, 3029 AK Rotterdam, The Netherlands; https://www.viroclinics.eu/). For the plaque reduction neutralization test (PRNT), a constant amount of virus (RSV-A2: VR-1540 or RSV-B: VR-1580) was mixed and pre-incubated with serial dilutions of sera or antibodies. Then monolayers of HEp-2 cells (HEp-2 cells, ATCC^®^ CCL-23™) were infected with the pre-incubated mixture of virus and neutralizing agent. After an incubation time of one day, cells were fixed and immune-stained with a murine monoclonal antibody directed against RSV F protein, followed by HRP-conjugated goat-anti-mouse antibody and TrueBlue (TB). The cell-wells were then scanned with the SX UV Analyzer (VC-M112). Spot counts (SC), or well area covered (WAC) by spots per well at each serum/antibody concentration were quantified by using the ImmunoSpot/BioSpot software (VC-M128). These values were used to calculate the dilution of serum/antibody that showed the selected reduction point (i.e., 50% for IC50).

### 2.8. Determination of IgE Reactivity to A2-G by Dot-Blot

Purified A2-G, BSA (negative control) and birch pollen allergen, Bet v 1 (positive control) were tested for IgE reactivity by non-denaturing dot blot assay. From each antigen, 0.5 µg was dotted onto nitrocellulose membranes (Schleicher & Schuell, Dassel, Germany), which were then blocked in gold buffer (50 mM sodium phosphate [pH 7.4], 0.5% (*v*/*v*) Tween-20, 0.5% (*w*/*v*) BSA, and 0.05% (*w*/*v*) sodium azide) and then incubated with 1:10 diluted sera from human subjects or buffer alone. After three washing steps with gold buffer, stripes were exposed overnight, at room temperature to 1 mL of ^125^I-labeled anti-human IgE antibodies (Demeditec, Kiel-Wellsee, Germany) diluted 1:10 in gold buffer. After an extensive washing step with gold buffer, stripes were dried and bound IgE antibodies were visualized by autoradiography using KODAK X-OMAT films (Kodak, Heidelberg, Germany) [32].

### 2.9. Statistical Analysis and Sequence Comparison

GraphPad Prism 6 Software was used for statistical analysis (GraphPad Software, San Diego, CA, USA). For comparison of antibody responses to the proteins (native A2-G, two denatured A2-G variants, deglycosylated A2-G and F0), the Mann Whitney U test was performed. Comparisons of IgG increases between different time points were performed by Wilcoxon’s matched-pairs signed rank test. In all cases, a *p* value < 0.05 was considered statistically significant. All statistical tests were non-parametric tests due to the small sample sizes. The correlations between antigen-specific IgG levels were calculated using the non-parametric, two tailed Spearman’s rank correlation coefficient (r_s_) method. P values below 0.05 were considered as significant. For sequence alignment, the Clustal Omega-multiple sequence alignment program (https://www.ebi.ac.uk/Tools/msa/clustalo/) was used.

### 2.10. Data Availability and Guidelines

The datasets generated and/or analyzed during the current study are available from the corresponding author on reasonable request. All experiments were performed in accordance with good laboratory practice guidelines of the Medical University of Vienna (https://www.meduniwien.ac.at/web/en/rechtliches/good-scientific-practice/).

## 3. Results

### 3.1. Design of an Expression Construct Corresponding to the Soluble Form of RSV A2-Derived G Protein and Synthetic Overlapping Peptides Spanning the Complete Protein

We designed a plasmid construct for the expression of the soluble form of the RSV A2-derived G protein in insect cells. The amino acid sequence of the complete RSV A2 has been aligned with subgroup A and B sequences in Figure 1a. The alignment demonstrates that A and B represent subgroups which differ substantially regarding their sequence but are very similar within each of the two subgroups. Accordingly, A2-derived G is representative for subgroup A. The expression construct for G used in our study was designed to remove the cytoplasmic domain and the hydrophobic transmembrane domain to facilitate its expression. The A2-G construct thus comprises the two mucin-like regions (black box: mucin-like region I; green box: mucin-like region II), which flank the conserved domain forming the disulfide-bond linked noose (green, four disulfide bonds) (Figure 1A,B). In the natural G protein, the two mucin-like regions contain several N- and O-linked glycans, as indicated in Figure 1B, upper part. The construct for expression in insect cells comprises the region from amino acid N66 to amino acid Q298 (Figure 1B, middle part). In total, 16 synthetic overlapping peptides with a length of 25 to 27 amino acids comprising the complete A2-derived G protein including its cytoplasmic and transmembrane domains with peptides GP1-GP4 were synthesized and purified (Figure 1A,B; Table 1). Thus, it was possible to use the peptides to discriminate antibody responses towards surface accessible (GP5–GP16) and buried parts (GP1–GP4) of the A2-derived G protein. We have also indicated in the peptides displayed in Table 1 N-(light green) and O-linked glycosylation sites (light blue), which allows discriminating peptides from glycosylated versus non-glycosylated regions in the natural protein as targets for antibodies.

### 3.2. Expression, Purification, and Characterization of Recombinant RSV A2-Derived G Protein

Recombinant RSV A2-derived G protein (i.e., A2-G) which was expressed in baculovirus-infected insect cells was secreted into the culture supernatant and could be purified by Nickel affinity chromatography under native conditions yielding approximately 1 mg pure protein per 20 mL of culture. Purified A2-G migrated in SDS-PAGE as a diffuse double band of approximately 40 and 50 kDa under reducing conditions, which specifically reacted with a monoclonal anti-His tag antibody indicating the presence of the hexahistidine tag at the C-terminal end of the protein (Figure 2). The fact that the apparent molecular weight observed by SDS-PAGE was greater than the molecular weight (i.e., 28.7 kDa with melittin signal peptide and 26.4 kDa without signal sequence), which was predicted according to the sequence, and the appearance of two bands can be explained by the presence of several glycosylation sites resulting in differently glycosylated versions of the recombinant protein. When SDS-PAGE was performed under non-reducing conditions, A2-G higher molecular weight bands were observed consistent with dimers, trimers, and tetramers of the protein (Figure 2, right part). The latter finding was confirmed by the analysis of A2-G by size exclusion chromatography (SEC) and dynamic light scattering (DLS) (Figure 3a,b,c). In the size exclusion chromatography experiment, A2-G appeared as one peak of approximately 32 kDa and one peak with approximately 70 kDa molecular weight (Figure 3a). In accordance with the SEC results, the DLS experiment also showed a peak of approximately 69 kDa, accounting for 96.5% of the mass and there was evidence for the presence of low amounts high molecular weight aggregates, which accounted for less than 4% of the mass (Figure 3b,c).

### 3.3. RSV G-Specific IgG Responses Dominate over IgM and IgA and Are Significantly Stronger than F0-Specific Responses

In a first set of experiments, we compared the IgG, IgM, and IgA responses of 18 healthy adult subjects to A2-G and F0 by ELISA (Figure 6). We found that IgG antibody levels specific for G (0.992, IQR 0.55–1.92) were significantly higher than F0-specific IgG levels (0.23, IQR 0.12–0.45), (*p* < 0.0001). Furthermore, G-specific IgG reactivity was significantly stronger than G-specific IgM (0.161, IQR 0.14–0.17) or IgA reactivity (0.17, IQR 0.10–0.25), (*p* < 0.0001) (Figure 6). We therefore focused on the further characterization of G-specific IgG responses.

### 3.4. Sequential RSV G-Derived Epitopes Are a Major Target of the IgG Response of Human Subjects

Figure 4 and Figure S1 shows the IgG reactivity of 12 healthy adult subjects with native recombinant A2-G, denatured A2-G versions, and deglycosylated A2-G in comparison with palivizumab-reactive A2-F0 and synthetic peptides spanning the complete A2-G protein. Sera were obtained from the subjects at several time points over the duration of one year, starting with January/February (Figure 4a), April/May (Figure 4b), September (Figure 4c), and January/February of the next year (Figure 4d). At each of the four timepoints, IgG levels to A2-G were significantly higher than IgG levels to F0 (Figure 4, Figure S1). Accordingly, we observed no correlation of IgG responses to F0 and native A2-G (Figure 5). A2-G-specific IgG was directed against the protein moieties and not against the carbohydrates because IgG levels to deglycosylated A2-G were slightly higher than native A2-G, indicating better accessibility of peptide epitopes after deglycosylation (Figure 4a). Interestingly, denaturation of G by SDS and heating as well as by SDS, the reducing agent TCEP and heating rather increased IgG reactivity as compared to native A2-G, indicating exposure of hidden or cryptic epitopes in the denatured proteins (Figure 4a–d). In fact, the heat map of IgG responses of individual subjects in Figure S1 shows that certain individuals (i.e., subjects 3, 4, 5, 6) reacted stronger with the two denatured A2-G version than with native A2-G and this was observed in serum samples obtained at each of the four time points. In agreement with the latter results, a highly significant correlation of IgG responses to native A2-G and denatured as well as to native versus deglycosylated A2-G was observed (Figure 5).

The subjects also showed IgG reactivity against the A2-G-derived synthetic peptides and thus recognized sequential, non-conformational epitopes (Figure 4, Figure S1). For six out of the 12 subjects (i.e., subjects 1, 2, 6, 8, 10, 12), the sum of peptide-specific IgG levels was higher than IgG levels to native A2-G, supporting the assumption that A2-G contains cryptic IgG epitopes (Figure S1). A trend towards a correlation of the sum of peptide-specific versus A2-G-specific IgG responses was found (Figure 5).

### 3.5. IgG Antibodies of Human Subjects Target Mainly Conserved and Exposed G-Derived Peptides but React also with Buried Peptides

The synthetic peptides spanning the complete A2-G protein included peptides (GP1-GP4) from the buried intracellular and transmembrane protein (Figure 1 and Figure 4, Table 1 and Figure S1), whereas the other peptides were part of the surface-exposed portion of G. Peptides GP9-GP12 comprised regions of the subgroups A and B that are conserved with GP10 representing the most conserved peptide, which also does not contain any glycosylation sites (Figure 1, Table 1). We found that the subjects displayed IgG reactivity not only against the surface-exposed peptides but certain among them (i.e., subjects 2, 5, 8 12) reacted also with peptides from the buried part of A2-G (Figure 4, Figure S1). However, peptides from the conserved region, in particular peptide GP10, which lacks glycosylation sites, were recognized by IgG antibodies from almost all subjects and with the highest IgG levels (Figure 4, Figure S1).

### 3.6. The IgG RSV G Epitope Recognition Profile of Human Subjects Is Scattered but Remains Constant over One Year

The IgG recognition of the analyzed subjects was focused on peptides derived from the central conserved A2-G domain (i.e., GP9–GP12), but at the same time IgG reactivity was scattered over the remaining portions of A2-G (Figure 4, Figure S1). Accordingly, we found no relevant correlations between the individual peptide-specific IgG responses with the A2-G-specific IgG responses, except for the highly conserved peptides GP9 and especially for GP10 but also for GP13 from the central region (Figure S2). Interestingly, the peptide IgG recognition profile of the whole group of subjects but also of the individual subjects did not change over a full year observation period during which serum samples had been taken at four different time points (Figure 4, Figure S1).

### 3.7. IgG Subclass Recognition of RSV G May Be Indicative of a Mixed Th1/Th2 Immune Response

Next, we analyzed the IgG subclass recognition of G and F0 by sera from 18 healthy adult subjects. Figure 7a–d show that the F0-specific IgG subclass reactivity was very weak, whereas A2-G was strongly recognized. In fact, for all four IgG subclasses, A2-G-specific reactivity was significantly higher than F0-specific reactivity (IgG_1_; IgG_2_: *p* < 0.0001; IgG_3_, IgG_4_: *p* < 0.001). The highest G-specific antibody levels were found for the IgG_1_ subclass (0.39, IQR 0.19–1.23), followed by IgG_2_ (0.12, IQR 0.08–0.16), (Figure 7). IgG_3_ (0.06, IQR 0.06–0.07) and IgG_4_ subclass (0.06, IQR 0.05–0.07) reactivity to G was low but detectable in a few subjects (Figure 7). We thus found an IgG subclass reactivity profile to G consisting mainly of IgG_1_ and IgG_2_ responses.

Nevertheless, we found that four subjects also displayed IgG_4_ subclass reactivity to A2-G, which may be indicative of a Th2 immune response (Figure 7). It has been reported earlier that certain subjects may mount RSV-specific IgE responses, which also indicates Th2 features of anti-RSV immune responses. We therefore tested adult subjects for IgE reactivity to nitrocellulose-dotted G and for control purposes the major birch pollen allergen Bet v 1 (positive control) and BSA (negative control); Figure S3. In fact, we found that two subjects (patient 7 and 9) exhibited weak but distinct IgE reactivity to G, whereas no signal was obtained to BSA and when buffer alone without serum was tested. Three of the subjects, who were allergic to birch pollen, showed IgE reactivity to the major birch pollen allergen, Bet v 1 (Figure S3).

### 3.8. Increases of IgG Responses to RSV G in Wheezing Children

Sera from well characterized children, who had experienced acute wheezing attacks were analysed [34,35]. In nasal swab tests of some of these children, RSV had been detected by PCR when they attended the clinic with the acute wheezing attack. Figure 8 shows the analysis of sera obtained from 12 RSV PCR-positive children at the time of the acute wheezing attack and of follow-up serum samples obtained approximately 11 weeks later for IgG reactivity to A2-G and F0. We found strong and significant increases of A2-G and F0-specific IgG antibodies when comparing baseline and follow-up sera (Figure 8) of children who had been tested positive for RSV by PCR. The increases of IgG levels were much stronger for G (acute visit: 0.13, IQR 0.0962–0.245; follow-up visit: 1.81, IQR 1.334–2.47) than for F0 (acute visit: 0.067, IQR 0.055–0.094; follow-up visit: 0.38, IQR 0.26–0.63), reaching optical density (OD) levels up to 3.0 for G in follow-up samples whereas F0-specific IgG levels increased not more than OD 1.5.

### 3.9. Sera Containing Mainly A2-G-Specific IgG Exhibit Virus-Neutralizing Activity

Figure 9 shows the IgG reactivity of the WHO-standard antiserum (Standard) and of a serum from an adult subject (Serum) to A2-G and recombinant palivizumab-reactive F0. Both sera showed strong and specific IgG reactivity to A2-G (Figure 9). Only weak (Standard) or no (adult serum) reactivity to F0 was observed (Figure 9) but when tested for reactivity with overlapping peptides spanning the A2-F0 protein, both sera showed IgG reactivity to a 25 amino acid long synthetic peptide (LGAIVSCYGKTKCTASNKNRGIIKT) from antigenic site IV of the fusion protein F (data not shown) [37,38].

Both, the standard and the serum as well as for control purposes, palivizumab, were then tested for virus neutralization using a plaque neutralization assay conducted for RSV-A2 (VR-1540) and RSV-B (VR-1580). We found that palivizumab and the standard could be diluted >256 times and still gave a 50% virus neutralization for RSV-A2 and RSV-B, whereas the serum yielded a 50% inhibition for RSV-B at the 1:256 dilution and could be diluted 1:152 for RSV-A to obtain a 50% inhibition. The negative control (monoclonal human IgG1) did not neutralize (Table 3).

## 4. Discussion

In this study, we expressed and purified the surface exposed portion of the RSV A2-derived surface glycoprotein as soluble and secreted protein and produced a set of 16 overlapping peptides spanning the complete A2-G, including its cytoplasmic and transmembrane part. We then investigated the RSV-specific IgG response in adult, RSV-exposed subjects as well as in a group of children who had experienced severe respiratory virus-induced wheezing attacks. Several results were obtained, which are of importance for the development of serological diagnostic tests for RSV infections and RSV-neutralizing vaccines. We found that IgG responses against G were much stronger than against a palivizumab-reactive complete recombinant F0 protein. This is in agreement with results obtained when natural F and G proteins were used for measuring human antibody responses [20] and indicates that G-based diagnostic assays for measuring RSV-specific IgG responses may be useful. Since RSV subgroups A and B differ mainly regarding the sequences of the G protein (Figure 1) whereas there is little variation among their fusion proteins, it may be possible to develop diagnostic tests based on G, which can discriminate subgroup A and B-specific antibody responses. In fact, our finding that the G-specific IgG responses in the subjects investigated in our study were mainly directed against sequential G-derived peptide epitopes indicates that it may be possible to develop serological tests based on subgroup A and B-derived peptides similar to what has been developed for the differential diagnosis of rhinovirus (RV) infections [35,39,40]. Our assumption that the G-specific IgG response is directed against non-conformational but rather sequential peptide epitopes is also supported by the finding that denatured and deglycosylated forms of A2-G showed similar and sometimes better IgG reactivity than native A2-G. Although our ELISA was not quantitative, we noted that the sum of peptide-specific IgG responses was sometimes higher than the G-specific IgG levels (Figure S1). Furthermore, our finding that G contains sequential peptide epitopes is in agreement with Fuentes et al., who demonstrated the presence of sequential peptide epitopes in G by phage display [25].

In fact, it has been possible to develop chips containing micro-arrayed peptides from the RV coat protein VP1, which allowed discriminating antibody responses against RV-A, RV-B, and RV-C species by serology [35]. Our data indicate that it may be possible to develop similar tests based on micro-arrayed peptides derived from the G proteins of RSV subgroup A and subgroup B capable of discriminating subgroup-specific antibody responses. The G peptide–specific IgG response was scattered over the sequence of G including also hidden peptides (i.e., cryptic epitopes), but the strongest IgG response was directed against the conserved, surface-exposed central region of G. The development of IgG response against buried peptides from the intracellular and transmembrane region may be explained by exposure of damaged cells expressing G protein and/or of damaged virus particles to the immune system. There may be several explanations for the preferential and higher IgG response against the surface exposed portions of G. First of all, this region is better accessible to antibodies on the intact virus and on the soluble G form. Second, GP10, the peptide eliciting the highest IgG responses did not contain glycosylation sites and hence was not hidden by carbohydrate moieties. Furthermore, repeated infections by different subgroups may enhance IgG responses mainly against the more conserved portions of the G protein because they are similar in the subgroups.

Interestingly, we found that the profile of IgG responses against the individual peptides did not change in samples obtained at four different time points during one year, which may indicate that adult subjects mount a constant secondary memory response against RSV epitopes. This is reminiscent of the constant IgG response observed for RV peptides in adult subjects, which is directed against one major epitope without changes of epitope specificity [41]. Likewise, the secondary IgE response of allergic subjects against individual allergens does not change once it has been constituted [42]. We therefore hypothesized that it may be possible to detect by serology increases of RSV-specific IgG responses as a result of a recurrent RSV infection. The measurement of increases of secondary antibody production is used in allergy to demonstrate allergen exposure in allergic subjects [43]. The same principle has been recently utilized to reveal RV infections as trigger factors for asthma and wheezing exacerbations. In fact, it has been shown in experimental RV infection models that exposure of adult asthmatics with RV induces asthma, which is associated with RV species-specific increases of antibody levels [44]. Likewise, it was shown that children who had experienced RV-induced wheezing attacks developed RV species-specific increases of IgG levels in follow up blood samples obtained approximately 11 weeks after RV-induced wheezing attacks [34,35]. Here, we show that also RSV-specific increases of IgG responses can be measured in RSV-PCR-positive children with such wheezing attacks in follow-up blood samples. Although there is no proof that the wheezing attack in these children was triggered by an RSV infection, our results encourage further investigations on if RSV-infections can be trigger factors for acute wheeze attacks. The increases of RSV-specific IgG antibodies in the children were detectable with F0 but G-specific antibody increases were stronger. Our results thus suggest that it may indeed be possible to develop serological tests, which can be used to investigate if RSV infections can be trigger factors for exacerbations of a variety of severe respiratory illnesses such as asthma and COPD in children but also in adults and elderly persons [1,4,40]. Accordingly, there is a need for serological tests to investigate this question because nucleic acid-based detection of virus can give false positive and false negative results, whereas serological tests based on the measurement of antibody increases may be more robust.

Our study has also provided results that are potentially important for understanding why natural RSV infections do not induce a protective immunity in everybody, and for the development of vaccines [2]. We found that the RSV-specific IgG response is scattered over the G protein and directed also against portions, which play no role regarding virus neutralization. For example, the strong IgG response of subject 2 against GP4 from the transmembrane domain is unlikely to have protective effects whereas IgG responses against the central conserved portion of G, which has been suggested to contain protective epitopes may contribute to virus neutralization [15,17,45,46,47]. Our finding that the RSV-specific IgG response is potentially mixed, resembling a Th1 type immunity involving potentially complement-activating IgG subclasses such as IgG_2_ and on the other hand Th2-like responses involving non-complement activationing IgG_4_ responses could have additional implications. It may be a starting point for further investigations trying to elucidate whether these different types of immune responses may be associated with different clinical courses of the infection. For example, it is quite possible that the production of complement activating IgG subclasses such as IgG_2_ [48] or the production of RSV-specific IgE and IgE-mediated allergic inflammation may be associated with a more severe course of the infection [49].

Interestingly we found that two sera, of which one is the WHO standard for RSV, exhibited strong virus-neutralizing activity and contained mainly IgG antibodies against A2-G but only weak or no responses against A2-F0. This finding is in agreement with recent studies, which reconsider the importance of RSV-G as a component of a neutralizing vaccine [18,48,50].

## 5. Conclusions

In summary, our study identifies RSV G and G-derived peptides as useful tools for serological diagnosis and investigation of RSV infections and to study RSV-triggered exacerbations of respiratory diseases. Furthermore, our data emphasize the importance of G for the development of RSV-neutralizing vaccines.

## Figures and Tables

**Table 1:** Sequences and characteristics of G-derived synthetic peptides (GP1-GP16). N-linked (green) and O-linked glycosylation sites (blue) are indicated. Shown are the sequences, lengths, molecular weights (MWs), and isoelectric points (pIs).

Peptide ID	Sequence	Length (AA)	MW (Da)	pI
GP1	MSKNKDQRTAKTLERTWDTLNHLLF	25	3047.4	9.69
GP2	KDQRTAKTLERTWDTLNHLLFISSC	25	2977.3	8.21
GP3	FISSCLYKLNLKSVAQITLSILAMI	25	2770.4	9.20
GP4	ILAMIISTSLIIAAIIFIASANHKV	25	2624.2	8.76
GP5	ANHKVTPTTAIIQDATSQIKNTTPT	25	2651.9	8.64
GP6	NTTPTYLTQNPQLGISPSNPSEITS	25	2660.8	4.00
GP7	SEITSQITTILASTTPGVKSTLQST	25	2564.8	5.72
GP8	STLQSTTVKTKNTTTTQTQPSKPTT	25	2680.9	10.30
GP9	SKPTTKQRQNKPPSKPNNDFHFEVF	25	2972.3	10.00
GP10	DFHFEVFNFVPCSICSNNPTCWAICKR	27	3178.6	6.72
GP11	TCWAICKRIPNKKPGKKTTTKPTKK	25	2857.5	10.49
GP12	KPTKKPTLKTTKKDPKPQTTKSKEV	25	2838.3	10.30
GP13	KSKEVPTTKPTEEPTINTTKTNIIT	25	2772.1	8.43
GP14	TNIITTLLTSNTTGNPELTSQMETF	25	2728.0	3.79
GP15	QMETFHSTSSEGNPSPSQVSTTSEY	25	2718.8	4.24
GP16	PSPSQVSTTSEYPSQPSSPPNTPRQ	25	2656.8	6.43

**Table 2:** Demographic characterization of study subjects.

Experiment	Number of Subjects	Gender Balance	Age Range	Mean Age	Allergic Patients	Non-Allergic Subjects
Figure 4, Figure 5, Figures S1 and S2	n = 12	m = 5, f = 7	26–46 (years)	32 (years)	n = 2	n = 10
Figure 6 and Figure 7	n = 18	m = 9, f = 9	23–59 (years)	40.4 (years)	n = 7	n = 11
Figure S3	n = 15	m = 7, f = 8	20–51 (years)	34.6 (years)	n = 8	n = 7
Figure 8	n = 12	m = 8, f = 4	6–34 (months)	19 (months)	unknown	unknown

**Table 3:** RSV-neutralizing titers of different samples.

Serum Sample	RSV-A2 (VR-1540)	RSV-B (VR-1580)
Standard	>256	>256
Patient serum	152.91	>256
Palivizumab	>256	>256
human IgG1	4.04	4.4

## Supplementary Materials

The following are available online at https://www.mdpi.com/2076-393X/8/2/337/s1, Figure S1: Heat map of IgG antibody responses (OD values corresponding to bound antibodies) of 12 adult individuals (#1–12) to recombinant, native G, G denatured by heat, SDS and TECEP, deglycosylated G, the individual G-derived peptides (GP1–GP16), recombinant F0 and the sum of peptide-specific IgG. Colors indicating different antibody levels are shown in the insert, Figure S2: Correlations of IgG levels specific for native G (x-axes) and G-derived peptides GP1–GP8 (a) and G-derived peptides GP9–GP16. (b) measured in sera from 12 adult individuals in individual scatter plots with Spearman correlation coefficient r and *p* values, Figure S3: IgE reactivity to nitrocellulose-dotted G protein and major birch pollen allergen, Bet v 1. Sera from 15 adult subjects (1–15) and buffer (B) without serum were tested for IgE reactivity to nitrocellulose-dotted A2-G (upper panel). BSA (middle panel) or recombinant major birch pollen allergen, rBet v 1 (lower panel). Bound IgE was detected with 125I-labeled anti-human IgE and visualized by autoradiography.
