# Supplementary material for: Features of the Human Antibody Response against the Respiratory Syncytial Virus Surface Glycoprotein G
Source: Vaccines (Basel). 2020 Jun 25;8(2):337. doi: 10.3390/vaccines8020337 (PMC7350215; doi:10.3390/vaccines8020337)
Supplement: Supplementary file 1 [file vaccines-08-00337-s001.pdf]

Serum samples taken between January and February

Pat. ID	GP1	GP2	GP3	GP4	GP5	GP6	GP7	GP8	GP9	GP10	GP11	GP12	GP13	GP14	GP15	GP16	∑ pept.	G	G denat.	G deglyc.	F0
#1	0.0693	0.0470	0.0654	0.0543	0.0901	0.0373	0.0552	0.1483	0.0499	0.0785	0.1429	0.2074	0.0683	0.0475	0.0489	0.0494	0.8703	0.4603	0.5146	0.5684	0.3426
#2	0.0912	0.0796	0.0711	1.1472	0.1167	0.0661	0.1037	0.0636	0.2104	0.1488	0.1553	1.1201	0.2780	0.0791	0.0756	0.0746	3.8177	2.5814	2.4656	2.5788	0.7649
#3	0.0495	0.0523	0.0423	0.0376	0.0580	0.0357	0.0604	0.0342	0.0709	0.1446	0.1298	0.1484	0.1308	0.0371	0.0409	0.0371	0.6244	1.7404	2.0315	2.1342	0.4703
#4	0.0605	0.0575	0.0600	0.0505	0.0510	0.0476	0.0833	0.0476	0.0723	0.1298	0.1952	0.1908	0.0545	0.0530	0.0545	0.0585	0.6715	1.1130	1.3910	1.4207	0.4172
#5	0.0598	0.0514	0.0453	0.0570	0.0710	0.0425	0.0598	0.0547	0.0822	0.1379	0.1467	0.2051	0.1150	0.0449	0.0472	0.0444	0.7580	1.2122	1.7561	1.7599	0.1486
#6	0.0887	0.1253	0.1091	0.1077	0.2396	0.0982	0.1424	0.0930	0.1390	0.2539	0.1680	0.1832	0.1020	0.1134	0.1177	0.0973	2.1787	1.1887	1.4137	1.4094	0.3668
#7	0.0654	0.0583	0.0752	0.0708	0.1586	0.0697	0.0888	0.0736	0.5711	0.3035	0.1842	0.2245	0.1642	0.0610	0.0616	0.0599	2.0696	2.7671	2.6516	2.6761	0.2474
#8	0.1630	0.2245	0.1734	0.2207	0.2004	0.1954	0.3055	0.1164	1.6857	0.3184	0.1775	0.1821	0.1742	0.2282	0.2390	0.2266	4.8310	2.1014	2.1097	2.1559	0.3114
#9	0.0668	0.0467	0.0639	0.0563	0.0553	0.0916	0.0744	0.0525	0.1049	0.1326	0.1507	0.1851	0.0525	0.0744	0.0539	0.0558	0.8806	0.9459	1.0742	0.7689	0.2027
#10	0.0838	0.0814	0.0833	0.0810	0.2259	0.0697	0.1003	0.0970	2.3660	0.3455	0.1897	0.2127	0.0838	0.0777	0.0890	0.0824	4.2689	2.4267	2.4154	2.4093	0.5949
#11	0.0655	0.0493	0.0549	0.0535	0.1347	0.0484	0.0789	0.0623	0.1706	0.2491	0.2020	0.2578	0.0489	0.1047	0.0498	0.0493	1.2633	1.5404	1.8227	1.8854	0.3814
#12	0.1794	0.2423	0.1689	0.2328	0.1770	0.1312	0.2418	0.1498	0.3330	0.2991	0.2695	0.3062	0.2166	0.2886	0.2938	0.2566	3.7865	1.6829	1.5365	1.8627	0.7079

Serum samples taken between April and May

Pat. ID	GP1	GP2	GP3	GP4	GP5	GP6	GP7	GP8	GP9	GP10	GP11	GP12	GP13	GP14	GP15	GP16	∑ pept.	G	G denat.	F0
#1	0.0747	0.0519	0.0538	0.0587	0.0747	0.0398	0.0582	0.1014	0.0543	0.0883	0.1674	0.2314	0.0796	0.0480	0.0446	0.0490	0.8173	0.5467	0.7737	0.7601
#2	0.0827	0.0642	0.0629	0.9515	0.0947	0.0568	0.0850	0.0564	0.1581	0.1544	0.1617	0.9233	0.2325	0.0749	0.0735	0.0661	3.0584	2.3796	2.0741	0.6729
#3	0.0517	0.0441	0.0436	0.0431	0.0426	0.0345	0.0599	0.0326	0.0656	0.2060	0.1542	0.1552	0.1202	0.0393	0.0374	0.0398	0.7013	1.4634	1.8331	0.3981
#4	0.0512	0.0516	0.0570	0.0588	0.0557	0.0431	0.0700	0.0436	0.0674	0.1338	0.1832	0.1823	0.0525	0.0498	0.0471	0.0579	0.6367	1.0530	1.3013	0.3592
#5	0.0671	0.0537	0.0495	0.0541	0.0888	0.0453	0.0685	0.0555	0.0814	0.1948	0.1573	0.1915	0.1212	0.0495	0.0500	0.0509	0.9705	1.0996	1.5363	0.1864
#6	0.1186	0.1294	0.1069	0.1153	0.1964	0.0961	0.1294	0.0994	0.1425	0.2428	0.1711	0.1931	0.1219	0.1472	0.1200	0.1153	2.2453	1.1179	1.4484	0.3276
#7	0.0559	0.0539	0.0693	0.0496	0.1151	0.0549	0.0660	0.0429	0.5229	0.2085	0.1329	0.1555	0.1637	0.0650	0.0568	0.0597	1.4990	2.3493	2.3941	0.3915
#8	0.1572	0.2600	0.1814	0.2631	0.2204	0.2436	0.3330	0.2019	1.7671	0.4563	0.1922	0.2220	0.2091	0.2677	0.2466	0.2631	5.4846	2.5075	2.5208	0.6839
#9	0.0606	0.0757	0.0844	0.0863	0.0810	0.0660	0.0946	0.0732	0.1741	0.2096	0.0786	0.1528	0.0820	0.0965	0.0883	0.0771	1.5202	1.8772	1.8457	0.8974
#10	0.0751	0.0604	0.0722	0.0788	0.1837	0.0576	0.0755	0.0590	2.2808	0.3078	0.1605	0.1468	0.0708	0.0718	0.0689	0.0736	3.6664	2.3823	2.4649	0.4797
#11	0.0607	0.0541	0.0645	0.0650	0.1281	0.0536	0.0802	0.0541	0.3108	0.3483	0.1732	0.2036	0.0508	0.0560	0.0550	0.0607	1.3093	1.4066	1.5110	0.4214
#12	0.1632	0.2349	0.1452	0.2487	0.1746	0.2145	0.2487	0.1528	0.3217	0.3331	0.2643	0.2914	0.1889	0.2733	0.2344	0.2572	3.7470	1.3686	1.2822	0.5604

Serum samples taken in September

Pat. ID	GP1	GP2	GP3	GP4	GP5	GP6	GP7	GP8	GP9	GP10	GP11	GP12	GP13	GP14	GP15	GP16	∑ pept.	G	G denat.	F0
#1	0.0652	0.0432	0.0496	0.0454	0.1111	0.0441	0.0711	0.3608	0.0738	0.0895	0.1282	0.1683	0.0882	0.0378	0.0409	0.0432	1.1563	0.4639	0.4886	0.7410
#2	0.0835	0.0763	0.0744	0.9158	0.1070	0.0595	0.0936	0.0638	0.1829	0.1474	0.1675	0.9034	0.2875	0.0730	0.0744	0.0797	3.2664	2.4744	2.2776	0.7421
#3	0.0451	0.0410	0.0423	0.0400	0.0548	0.0391	0.0562	0.0350	0.0589	0.1721	0.1197	0.1289	0.1082	0.0414	0.0433	0.0396	0.5289	1.3380	1.6938	0.3848
#4	0.0520	0.0428	0.0483	0.0437	0.0437	0.0456	0.0672	0.0474	0.0704	0.1155	0.1721	0.1464	0.0479	0.0446	0.0456	0.0469	0.5717	0.9353	1.1153	0.3300
#5	0.0639	0.0455	0.0469	0.0432	0.1209	0.0418	0.0704	0.0474	0.0869	0.2304	0.1223	0.1568	0.1494	0.0432	0.0478	0.0428	0.9371	1.4020	1.2801	0.2166
#6	0.1171	0.1318	0.1147	0.1294	0.2721	0.1124	0.1697	0.1062	0.1384	0.2816	0.1427	0.1446	0.1237	0.1536	0.1446	0.1275	2.4101	1.1505	1.1230	0.3755
#7	0.0609	0.0550	0.0698	0.0634	0.0968	0.0560	0.0663	0.0481	0.5119	0.2270	0.2004	0.1498	0.1553	0.0703	0.0604	0.0526	1.5476	2.4747	2.4963	0.4446
#8	0.2234	0.2669	0.2689	0.3109	0.2784	0.2914	0.4299	0.2199	1.5695	0.4404	0.1859	0.2299	0.2559	0.3514	0.3409	0.3389	6.0027	2.4078	2.1659	0.7498
#9	0.0788	0.0829	0.0929	0.0743	0.0734	0.0689	0.0906	0.0657	0.2129	0.2949	0.1332	0.1395	0.0684	0.0847	0.0802	0.0861	1.7274	1.6517	1.9530	0.9998
#10	0.0702	0.0735	0.0778	0.0654	0.2158	0.0673	0.1008	0.0645	2.2515	0.3486	0.2177	0.1714	0.0616	0.0764	0.0726	0.0778	3.8869	2.3961	2.4902	0.5066
#11	0.0707	0.0599	0.0653	0.0658	0.1504	0.0554	0.0901	0.0513	0.2846	0.4152	0.1919	0.1522	0.0576	0.0662	0.0716	0.0712	1.6951	1.8050	1.8037	0.4553
#12	0.1696	0.2409	0.1274	0.2179	0.1777	0.2280	0.2788	0.1677	0.3396	0.3727	0.2544	0.2797	0.2007	0.3061	0.2524	0.2764	3.8900	1.4356	1.4921	0.6098

Serum samples taken between January and February (the following year)

Pat. ID	GP1	GP2	GP3	GP4	GP5	GP6	GP7	GP8	GP9	GP10	GP11	GP12	GP13	GP14	GP15	GP16	∑ pept.	G	G denat.	F0
#1	0.0716	0.0628	0.0697	0.0653	0.0930	0.0429	0.0604	0.1340	0.0531	0.1057	0.1354	0.2051	0.0872	0.0453	0.0477	0.0477	0.9670	0.5052	0.6820	0.7867
#2	0.0921	0.0639	0.0558	0.9373	0.0944	0.0563	0.0754	0.1517	0.1579	0.1293	0.1579	0.7871	0.2414	0.0701	0.0758	0.0649	2.9704	2.4118	2.2038	0.6120
#3	0.0530	0.0467	0.0458	0.0592	0.0418	0.0369	0.0565	0.0369	0.0588	0.1656	0.1304	0.1367	0.0975	0.0418	0.0383	0.0387	0.5301	1.2766	1.5962	0.3138
#4	0.0484	0.0488	0.0426	0.0448	0.0448	0.0466	0.0663	0.0412	0.0587	0.1031	0.1344	0.1425	0.0511	0.0444	0.0453	0.0493	0.4464	0.8031	0.9470	0.1873
#5	0.0464	0.0460	0.0432	0.0446	0.0706	0.0381	0.0595	0.0446	0.0664	0.1477	0.1291	0.1231	0.0873	0.0409	0.0437	0.0441	0.6243	0.9387	1.4208	0.1510
#6	0.1345	0.1586	0.1255	0.1435	0.2302	0.1302	0.1705	0.1094	0.1359	0.2818	0.1530	0.1695	0.1307	0.1705	0.1421	0.1402	2.5260	1.0305	0.9760	0.3405
#7	0.0455	0.0418	0.0438	0.0434	0.0649	0.0393	0.0562	0.0393	0.4308	0.3229	0.1732	0.1133	0.1220	0.0451	0.0434	0.0401	1.1623	2.0508	2.0620	0.3862
#8	0.1990	0.2989	0.1990	0.2893	0.2228	0.2852	0.3250	0.2237	1.4047	0.4323	0.1618	0.2223	0.2081	0.3296	0.2856	0.2925	5.3799	2.0928	2.1579	0.6460
#9	0.0556	0.0616	0.0769	0.0732	0.0579	0.0546	0.0658	0.0685	0.2802	0.2871	0.1519	0.1417	0.0611	0.0847	0.0593	0.0866	1.3165	1.8819	1.3776	0.9947
#10	0.0599	0.0582	0.0573	0.0591	0.0976	0.0503	0.0766	0.0612	1.9910	0.2323	0.1824	0.1522	0.0525	0.0525	0.0525	0.0612	2.7321	2.1581	2.2789	0.4458
#11	0.0685	0.0568	0.0643	0.0638	0.1286	0.0508	0.0783	0.0536	0.2283	0.2865	0.2096	0.2418	0.0531	0.0517	0.0498	0.0568	1.2416	1.2723	1.4675	0.3052
#12	0.2243	0.2511	0.1644	0.2276	0.1984	0.1856	0.2931	0.1753	0.3901	0.3095	0.2615	0.3185	0.2247	0.2379	0.2393	0.3072	4.0085	1.3088	1.3201	0.6408

< 0.065	
0.065–0.1	
0.1–0.3	
0.3–0.9	
> 0.9	

Figure S1. Heat map of IgG antibody responses (OD values corresponding to bound antibodies) of 12 adult individuals (#1–12) to recombinant, native G, G denatured by heat, SDS and TECEP, deglycosylated G, the individual G-derived peptides (GP1–GP16), recombinant F0 and the sum of peptide-specific IgG. Colors indicating different antibody levels are shown in the insert.

a

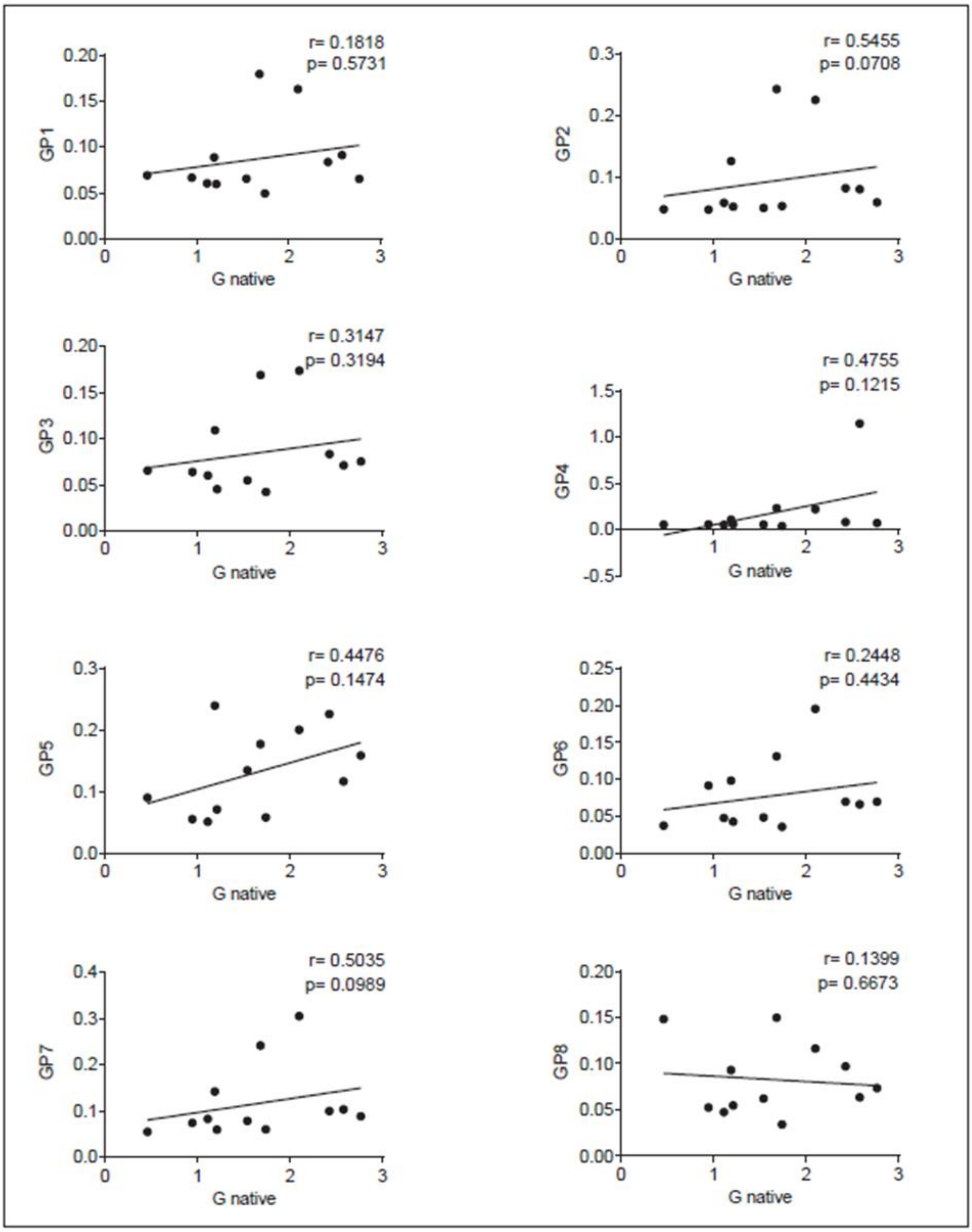
r= 0.1818
p= 0.5731
GP1
G native
r= 0.5455
p= 0.0708
GP2
G native
r= 0.3147
p= 0.3194
GP3
G native
r= 0.4755
p= 0.1215
GP4
G native
r= 0.4476
p= 0.1474
GP5
G native
r= 0.2448
p= 0.4434
GP6
G native
r= 0.5035
p= 0.0989
GP7
G native
r= 0.1399
p= 0.6673
GP8
G native

b

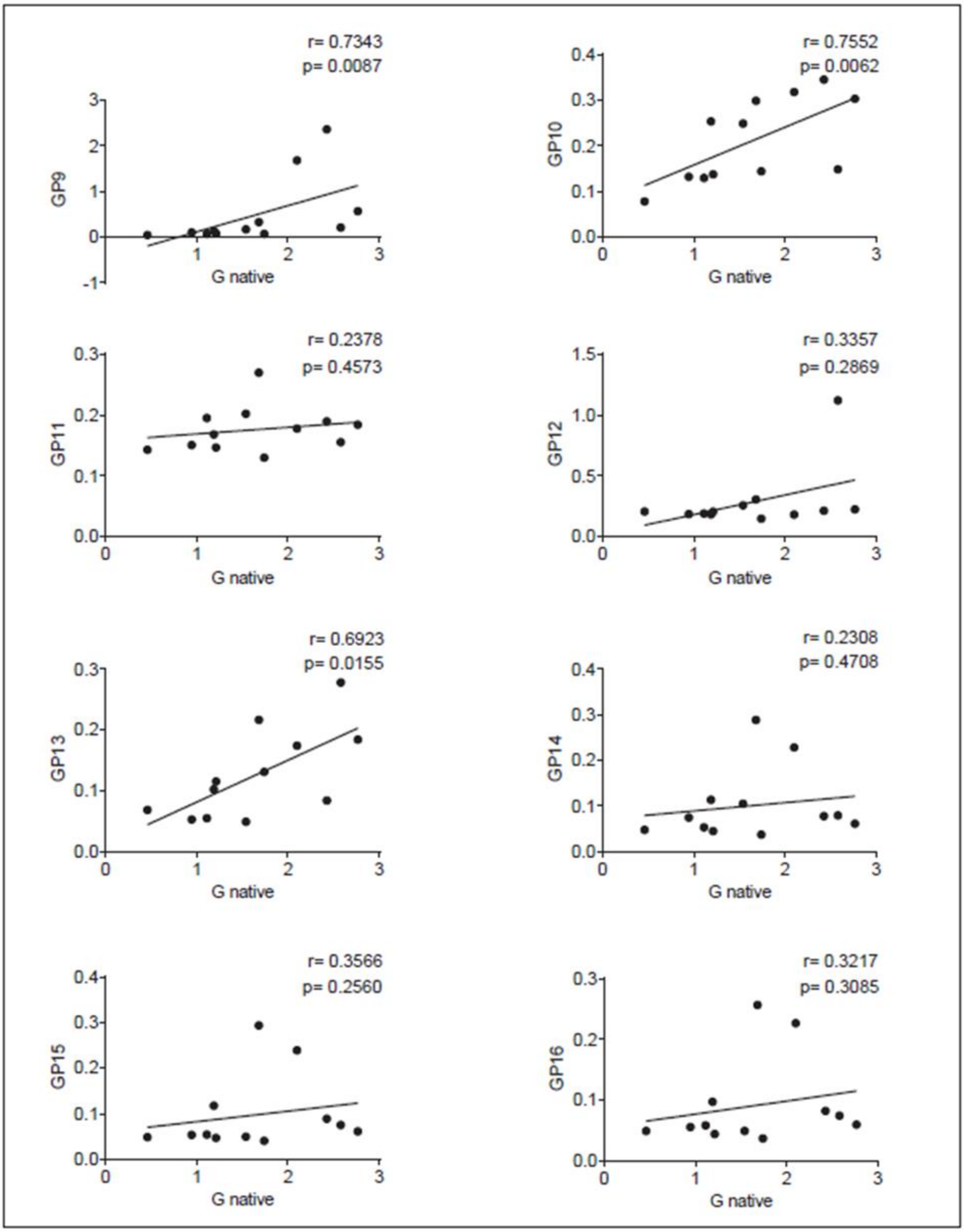

Figure S2. Correlations of IgG levels specific for native G (x-axes) and G-derived peptides GP1–GP8 (**a**) and G-derived peptides GP9–GP16. (**b**) measured in sera from 12 adult individuals in individual scatter plots with Spearman correlation coefficient r and *p* values.

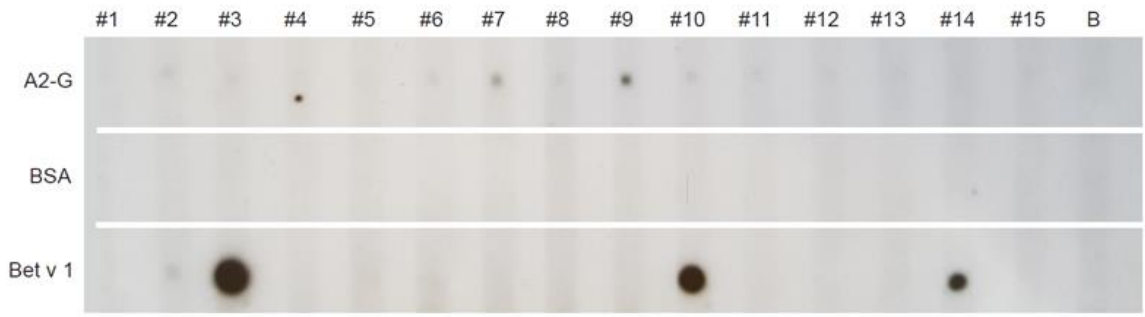

Figure S3. IgE reactivity to nitrocellulose-dotted G protein and major birch pollen allergen, Bet v 1. Sera from 15 adult subjects (1–15) and buffer (B) without serum were tested for IgE reactivity to nitrocellulose-dotted A2-G (upper panel). BSA (middle panel) or recombinant major birch pollen allergen, rBet v 1 (lower panel). Bound IgE was detected with ^{125}I-labeled anti-human IgE and visualized by autoradiography.
